# Stepping in Place While Voluntarily Turning Around Produces a Long-Lasting Posteffect Consisting in Inadvertent Turning While Stepping Eyes Closed

**DOI:** 10.1155/2016/7123609

**Published:** 2016-08-22

**Authors:** Stefania Sozzi, Marco Schieppati

**Affiliations:** ^1^Fondazione Salvatore Maugeri (IRCCS), Centro Studi Attività Motorie, Via Salvatore Maugeri 10, 27100 Pavia, Italy; ^2^Department of Public Health, Experimental and Forensic Medicine, University of Pavia, Via Forlanini 2, 27100 Pavia, Italy

## Abstract

Training subjects to step in place on a rotating platform while maintaining a fixed body orientation in space produces a posteffect consisting in inadvertent turning around while stepping in place eyes closed (podokinetic after-rotation, PKAR). We tested the hypothesis that voluntary turning around while stepping in place also produces a posteffect similar to PKAR. Sixteen subjects performed 12 min of voluntary turning while stepping around their vertical axis eyes closed and 12 min of stepping in place eyes open on the center of a platform rotating at 60°/s (pretests). Then, subjects continued stepping in place eyes closed for at least 10 min (posteffect). We recorded the positions of markers fixed to head, shoulder, and feet. The posteffect of voluntary turning shared all features of PKAR. Time decay of angular velocity, stepping cadence, head acceleration, and ratio of angular velocity after to angular velocity before were similar between both protocols. Both postrotations took place inadvertently. The posteffects are possibly dependent on the repeated voluntary contraction of leg and foot intrarotating pelvic muscles that rotate the trunk over the stance foot, a synergy common to both protocols. We propose that stepping in place and voluntary turning can be a scheme ancillary to the rotating platform for training body segment coordination in patients with impairment of turning synergies of various origin.

## 1. Introduction

When walking in everyday environments, subjects frequently change direction to negotiate corners and avoid obstacles. The ability to change direction and the ability to accurately control the curved trajectory while walking are essential components of successful navigation. Under the curved walking condition, the control of the muscle synergies takes into account not only the obligatory propulsion but also the equilibrium constraints connected to body rotation. Turning involves complex orientation of head, trunk, pelvis, and feet [1–5] and is accompanied by trunk inclination to the inner part of the trajectory to counteract the centrifugal acceleration acting on the walking body [1, 6, 7]. Also, motion of the lower limbs is asymmetric, whereby the leg inside the trajectory travels a shorter pathway than the outside leg [1–6]. Not unexpectedly, given the complex coordination and multisensory integration underlying curved walking [8], studies requiring subjects to travel both linear and circular pathways have detected abnormalities in patients with neurological disorders [9–13].

Rehabilitation of curved walking has been advocated by several investigators [7, 14, 15], and preliminary data on the potentially positive effect of circular treadmill training on curved walking in PD patients are available [16]. The improvement of the velocity of curved walking in these patients would possibly rest on the training of the neural circuits subserving the complex synergies for turning mentioned above.

The nervous system can learn to produce curved walking. Evidence thereof is represented by the so-called podokinetic after-rotation (PKAR). Previous studies showed that, after prolonged stepping in place on a rotating platform, subjects asked to walk normally on firm floor straight ahead without vision unknowingly generated a curvilinear path [14, 17]. In addition, when subjects were asked to step in place without vision after having stepped on the rotating platform for a prolonged period, they continued to rotate in the horizontal plane around their vertical axis for a while after the halt of the platform [18–20]. This PKAR has been considered the effect of adaptation to the continuous perturbation of the foot position by the podokinetic stimulation (produced by the rotation of the platform upon which subjects step while keeping the orientation of trunk and head fixed relative to space) [20].

Thus, the podokinetic stimulation produces a rotation of the feet below the head and trunk, the orientation of which hardly changes with respect to the environment. The foot rotation is then counteracted by a corrective repositioning action, since the foot is moved to its original position again, in a direction opposite to the direction of the rotating platform, so that body orientation in space stays unchanged. Subjects are focused on maintaining stable head and trunk and rotate their feet back to the original position almost unconsciously, thereby neutralizing the effect of the platform rotation on the upper body. Then, when the platform is stopped but the subjects are asked to continue stepping in place, subjects continue turning in the same direction in which they rotated the feet during the counteraction that replaced them in the “right” position, necessary for keeping their body position fixed in space. Remarkably, such PKAR is not consciously perceived [14, 18]. Of note, a visual or haptic input given for few seconds during the PKAR period can reduce the PKAR velocity; when the new information is removed, the PKAR reappears [21].

Inadvertent rotation while stepping in place is not an odd or peculiar effect. Similar body rotation effects are obtained by the unilateral vibration of neck and trunk muscles while walking or stepping in place ([22, 23] and see [24]). Vibrating the sternocleidomastoid muscle, for example, compels the body to turn to the side opposite to vibration [23]. Moreover, rotation posteffects have been observed. A prolonged optokinetic stimulation causes a consistent posteffect. After that stimulation, blindfolded subjects turned around when attempting to step in place without turning [25]. Under the above conditions, as well as with PKAR, subjects were not aware of any body rotation while stepping in place with vibration or after optokinetic stimulation.

We put forward the hypothesis that a podokinetic after-effect can take place after voluntary turning while stepping in place, that is, in the absence of the stimulation produced by the rotating platform. There is no quantitative information to date on the events occurring after a period of prolonged stepping in place while turning around the body's vertical axes. Contrary to what happens while stepping in place on the rotating platform, during voluntary turning subjects are certainly aware of the deliberate rotation of their feet in the direction they want to turn in. Moreover, head and trunk are not fixed in space but rotate continuously, and vision is removed in order to annul eye movements and optokinetic effects.

## 2. Methods

### 2.1. Subjects and Tasks

Sixteen healthy subjects (7 males and 9 females, mean age 27.5 yrs ± 6.4 SD, height 173.4 cm ± 7.9 SD, and weight 67.9 kg ± 10.15 SD) participated in the experiments. All subjects were naïve to the experimental procedure and all succeeded in performing the trials without difficulty. Experiments were performed in accordance with the Declaration of Helsinki. The ethics committee had approved the experiment (Central Ethics Committee, Fondazione Salvatore Maugeri, approval number 806 CEC). All procedures were carried out with the adequate understanding and written informed consent of each subject.

Subjects performed a trial in which they stepped in place with bare feet, eyes open, fixing a target at eye level at a distance of about three meters, at the centre of a disc of 2 m of diameter, rotating at a velocity of 60°/s in the counterclockwise direction for 12 min, thereby inducing a repetitive podokinetic stimulation. During this period, subjects maintained a roughly constant position of the body in space. They stepped at their own cadence, without any imposed cue. Following the 12 min period on the rotating platform, the platform was stopped. Subjects wore an eye-mask on the forehead during the podokinetic stimulation and lowered it at eye level to block vision when the platform stopped. Then they were told to continue stepping in place for at least 10 min more.

In another trial, blindfolded subjects voluntarily turned around while stepping in place at their natural cadence and at their preferred angular velocity on a stationary surface (the same platform, motionless) for 12 min. After this period, an operator asked the subject to stop turning and continue stepping in place for at least 10 min more. During voluntary turning, subjects were asked to turn in place in clockwise direction. In this way, during the posteffect of the podokinetic stimulation and of the voluntary turning, blindfolded subjects rotated in the same direction while stepping. Subjects did not practice stepping prior to recording. The platform rotation and the voluntary rotation trials were performed in a different day and were randomized across subjects.

Under both conditions (podokinetic stimulation and voluntary turning) subjects stepped inside a plastic hula-hoop of 50 cm of diameter, loosely fixed at pelvic height by elastic straps secured to the platform outer railing. This hula-hoop prevented subjects' displacement from the platform rotation centre while stepping in place, in particular with eyes closed (during voluntary turning condition and the posteffect periods). Lightly touching the hoop with the pelvis occurred from time to time, but this gave no cue regarding the position in space, during either the rotation or the posteffect, as shown from the participants' report at the end of the experiments. Subjects' arms were folded under both conditions. Of note, no safety harness was employed nor did subjects hold onto a stable overhead [20, 26] or otherwise firm external structure.

### 2.2. Data Acquisition and Analysis

In order to capture both rotation in space and the feet stepping movements, eleven reflective markers were placed bilaterally on the following body positions: three markers were mounted on a light inner frame of a helmet in correspondence with vertex and lateral head position, and the others were placed on the acromion, lateral malleolus, posterior heel, and forefoot (dorsally, about over the 1st metatarsophalangeal joint). Kinematic data were recorded by means of a device (Smart-D, BTS, Italy) composed of 12 optoelectronic cameras, at a sampling frequency of 100 Hz, and stored in a PC. The marker traces were filtered with a third-order low pass Butterworth filter with a cut-off frequency of 1.5 Hz (software developed in MATLAB, MathWorks Inc., USA). This frequency was chosen based on the frequency spectrum of the trace of shoulders marker displacement, which showed no frequency content >1.1 Hz in any subject. Off-line analysis was performed on the data acquired in a time-window that started 2 min before the platform stop, or 2 min before the signal to stop the voluntary turning, and lasted from 10 to 15 minutes.

**Figure 1:**
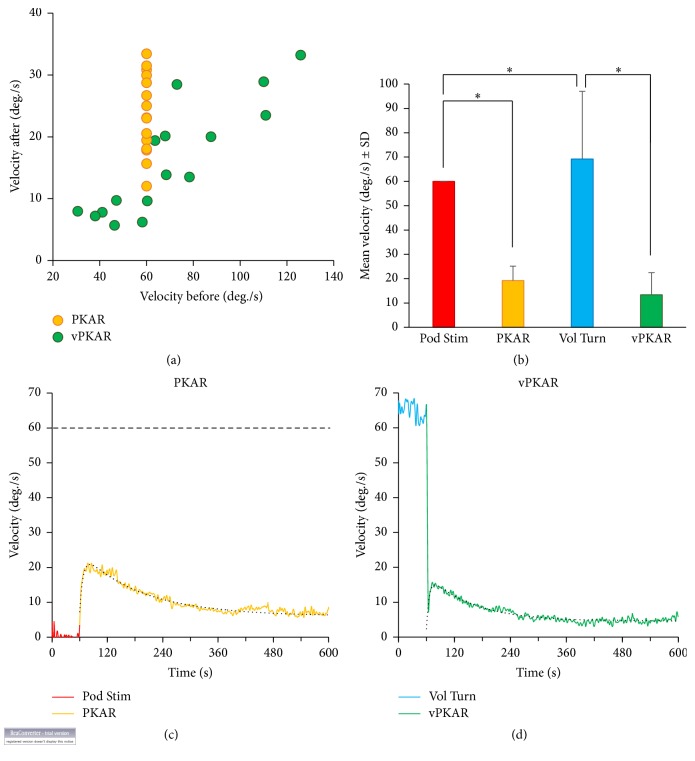
Posteffect of podokinetic stimulation and voluntary turning. In (a) the peak rotation velocity for each subject during the posteffect is plotted against the rotation velocity during the two conditioning procedures. (b) shows the mean angular velocity of the platform (Pod Stim) and of the body across subjects, during conditioning and posteffects. (c) shows the mean trace of the velocity of the body rotation (obtained by averaging the traces of all subjects) during the last minute of the podokinetic stimulation (Pod Stim, red colour, from 0 s to 60 s) and during the immediately following podokinetic after-rotation (PKAR, yellow color, 60 s to 600 s). The horizontal dashed line indicates the platform rotation velocity. (d) shows the angular velocity during the last part of voluntary turning (Vol Turn, blue, 0 s to 60 s) and the posteffect (vPKAR, green, 60 s to 600 s). The mean angular body velocity was almost null during Pod Stim but was more than 60°/s during Vol Turn (compare (c) and (d)). During the two posteffects, the mean velocities were just larger for PKAR compared to vPKAR but showed a similar initial rise and decay (the black dotted lines are the exponential fit). *∗* indicates significant difference (*p* < 0.05) between mean velocities.

For each trial of each subject, a software program developed in MATLAB calculated the angle described in the horizontal plane by the line-segment joining the markers placed on the shoulders within each 10 ms time interval (defined by the sample frequency). This was taken as the body rotation angle. The cumulative angle described by the body was calculated as the sum of the successive angles for the entire duration of the acquired epochs. The instantaneous angular velocity of the body rotation was the numeric derivative of the cumulative angle. A similar calculation was made for the angular rotation of the head, based on the recording of the two lateral markers placed on the helmet frame. The body angular velocity was then filtered with a low pass filter with a cut-off frequency of 2 Hz, just in order to clearly display the time course of the posteffects in Figure 1. From the head angular velocity (not filtered), we calculated the angular acceleration of the head rotation in the horizontal plane. The mean angular acceleration of the head was then obtained by averaging the rectified trace of the angular acceleration in the last minute of voluntary turning or stepping in place on the rotating platform and in a time period of one minute around the maximum peak of the rotation velocity during the two posteffects. The mean peak acceleration was also computed.

In order to estimate the time course of the posteffects induced by the stepping in place on the rotating platform or induced by the voluntary turning, the trace of shoulder rotation velocity in the postperiod was fitted with an exponential function *y* = *Ae* ^−*t*/*τ*_1_^ + *Be* ^−*t*/*τ*_2_^ + *C*. Based on visual checking of the data and on previously published analyses [21, 27–29], a function characterized by two time constants was chosen in order to describe the initial rise in the posteffect angular velocity, which is then followed by a slow decay over time. To this aim, the iterative conjugate gradient method of the Excel® Solver Utility was used, *τ*_1_ and *τ*_2_ being the time constants, *C* being the asymptotic value of the function, and *A* + *B* + *C* being the intercept with the ordinate. The values of *A*, *B*, *C*, and *τ*_1_ and *τ*_2_ parameters were computed by using the minimum sum squared algorithm. The maximum value of the double-exponential function was assumed as the peak rotation velocity reached in the posteffect. The time at which the posteffects disappeared was estimated by 3*∗τ*_2_, because at this time the rotation velocity has dropped to 5% of its peak value. The mean angular velocity of body rotation during voluntary turning was calculated in the last minute of this task before subjects were told to stop turning and continue to step in place. The rotation velocity while stepping on the rotating platform was simply the platform rotation velocity, since head and shoulders did not actually rotate in space while stepping on the rotating platform.

The time-relationship between head and shoulder rotation was computed while stepping on the rotating platform and thereafter and during voluntary turning and thereafter. The time lag was obtained by the cross-correlation analysis. To this aim, the filtered traces (high-pass filter with a cut-off frequency of 0.1 Hz) of the cumulative angles described by shoulder girdle and head axes on the horizontal plane were used. The time lag was the time interval at which the absolute value of the cross-correlation coefficient (*R*) was maximum. A negative time lag indicated that shoulders lagged behind the head movement.

Cadence, height reached by the feet (marker placed on lateral malleolus) during the swing phase, and duration of the stance phase (the time interval between the lowermost malleolus position and the subsequent malleolus off) were calculated by software developed in LabVIEW (National Instruments Corporation, Austin, TX). For each subject, the mean cadence, height of feet, and duration of stance phase were calculated within the last minute of the period of voluntary turning or stepping on the rotating platform and within one minute around the peak of velocity during the two posteffects. Further, by using the markers placed on the heel and forefoot, the step yaw angle of the foot of the side corresponding to the direction of rotation was calculated for each condition and subject by software developed in MATLAB.

### 2.3. Statistics

A 2-way repeated-measure ANOVA with experimental condition (podokinetic stimulation or voluntary turning) and pre- and posteffects (PKAR or vPKAR, i.e., the PKAR following voluntary turning) as factors was used to compare the following: rotation velocity of shoulders, head velocity and acceleration, time lags between head and shoulder axis rotations, and cadence and step angle. The time constants (*τ*_1_ and *τ*_2_) of the shoulder axis angular velocity during the two posteffect periods were compared by a 2-way repeated-measure ANOVA, with time constants and posteffect of voluntary turning (vPKAR) or podokinetic stimulation (PKAR) as factors. The duration of the stance phase and the height of foot lifting were compared by a 3-way repeated-measure ANOVA with conditioning procedure (voluntary turning or podokinetic stimulation), pre- and posteffects, and feet as factors. For all ANOVAs, the post-hoc test analyses were made with Fisher's LSD test. The software package used was Statistica (StatSoft, USA).

## 3. Results

### 3.1. Posteffect of Podokinetic Stimulation and of Voluntary Turning

After the 12 min period of stepping in place on the rotating platform, subjects showed a clear-cut PKAR. All the subjects, when the platform was stopped and they were asked to continue stepping in place, went on inadvertently turning around in the same direction as their feet had rotated to counteract the platform rotation (i.e., opposite to the direction of the platform movement). Likewise, after the 12 min period of voluntary stepping and turning, all subjects showed a posteffect (a PKAR following* voluntary* stepping and turning, vPKAR) and continued turning in the same direction as the direction of the voluntary rotation. This posteffect was broadly similar to that observed after the podokinetic stimulation. No subject, interviewed at the end of the experiment, reported any perception of turning during PKAR or during the vPKAR. For each subject and condition, the angular velocity recorded during the posteffect was fitted with a double-exponential function, where *τ*_1_ described the initial rise and *τ*_2_ described the decay of the rotational posteffect. The highest value of the function was the peak rotation velocity reached during the posteffect. There was a remarkable analogy in the time constants and peak velocities between the two protocols, both within and across subjects.

Figure 1(a) shows the angular velocities of the rotating body (each symbol corresponds to one subject) observed in the postperiod (after the platform rotation or after the voluntary turning, inordinately), plotted against the corresponding angular velocities observed during platform rotation or during voluntary turning. The platform rotation velocities were identical for all subjects (60°/s, red bar in Figure 1(b)), while the velocities in the after-period (PKAR) peaked in a range from about 10°/s to 35°/s. The mean value across subjects was 19.2 ± 5.9°/s (Figure 1(b), yellow bar). Therefore, there was a mean reduction to about 30% with respect to the velocity of the platform. During voluntary turning (Figure 1(a)), the velocity of body rotation was largely different across subjects (they were free to select their velocity of turning while stepping), ranging from about 30°/s to about 120°/s. The mean value was 69.2°/s ± 27.7 (Figure 1(b), blue bar). After the period of voluntary turning, when subjects were asked to continue stepping in place without deliberately turning around, they continued to rotate in the same direction as that of the preceding voluntary rotation, with a mean velocity of 13.4°/s ± 9.7 (Figure 1(b), green bar). Clearly (Figure 1(a)), the velocity of rotation in the posteffect was proportional to the velocity of voluntary turning. On average, the vPKAR had an angular velocity of about 20% of the mean velocity during the voluntary turning.

ANOVA showed no significant difference in the mean angular velocity between voluntary turning and podokinetic stimulation (main effect, *F*(1,15) = 0.12, *p* = 0.72). In the posteffects, the angular velocity significantly decreased with respect to that during both voluntary turning and podokinetic stimulation (pre- versus posteffect, *F*(1,15) = 245.86, *p* < 0.01). However, there was a significant interaction between conditions and pre- and posteffects (*F*(1,15) = 11.1, *p* < 0.01), since the mean angular velocity during voluntary turning was 15% greater than during platform rotation (post-hoc test, *p* < 0.05), while during vPKAR the mean angular velocity was smaller (even if not significantly so, post-hoc test, *p* = 0.09) than during PKAR.

Because the range of velocities during voluntary turning was large, the comparison was also done directly for the few participants that had voluntary turning velocities (6 subjects, mean velocity 65.26°/s ± 5.6) very close to that of the platform rotation. There were no difference in the angular velocities between voluntary turning and podokinetic stimulation (*F*(1, 5) = 0.3, *p* = 0.61), a significant difference between pre- and posteffect (*F*(1, 5) = 692.24, *p* < 0.01), and an interaction between condition and pre- and posteffect (*F*(1, 5) = 98.83, *p* < 0.01). The interaction was due to the significant difference between PKAR and vPKAR (post-hoc test, *p* < 0.01), since the angular velocity (13.3°/s ± 8.2) was smaller during vPKAR compared to PKAR (20.9°/s ± 5.1). Thus, there was a difference between the two conditions in terms of the posteffect relative to the preeffect.

Figure 1 also shows the time course of the posteffects. The mean trace of angular rotation velocity over time (all subjects' traces averaged) during the PKAR (c) and the vPKAR (d) is reported. In both cases, subjects briefly ceased turning for a moment (lasting less than 5 s, not obvious in the figure) when the platform stopped or at the end of voluntary turning, when they were told to continue stepping without turning. Then, they resumed stepping and turning around (involuntarily). In both cases, turning velocity rapidly increased to a maximum value, usually peaking in the first min or so. Next, the angular velocity slowly decreased until the end of the acquisition period.

**Figure 2:**
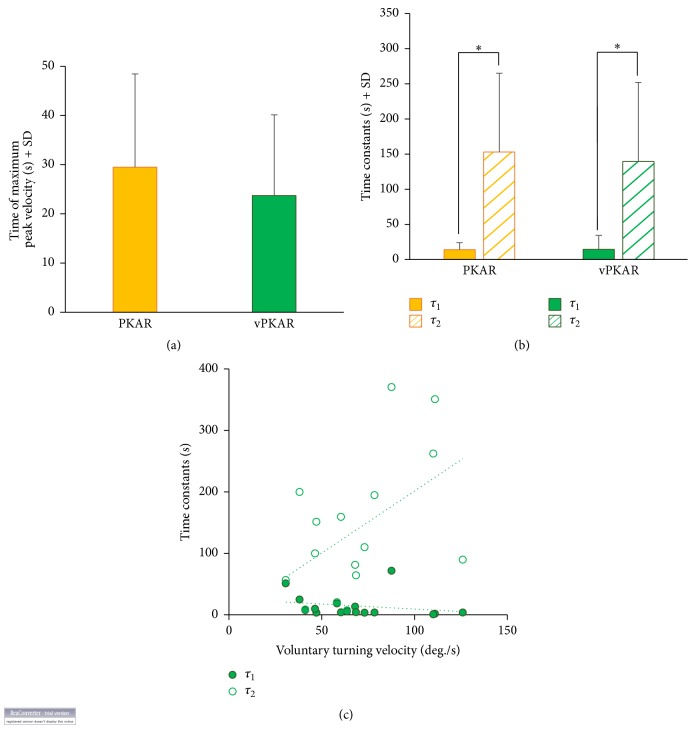
Peak velocity and time course of the posteffects. (a) Time at which rotation velocity peaked during PKAR (yellow bar) and during the posteffect of voluntary turning (vPKAR, green bar). (b) Time constants of the exponential fit to the time course of the two posteffects. *τ*_1_ was the time constant of the initial rise and *τ*_2_ was the time constant of the decay in angular velocity over time. (c) For each subject, the time constants (*τ*_1_ green filled symbols, *τ*_2_ open symbols) of vPKAR are plotted against the rotation velocity of voluntary turning. *∗* indicates significant difference (*p* < 0.05) between mean time constants (b).

Figure 2(a) shows that the time at which the maximum angular velocity was reached during the posteffects was similar for both conditions (29.5 s ± 18.9 for PKAR and 23.7 s ± 16.4 for vPKAR,* t*-test, *p* = 0.37).  Figure 2(b) shows the mean values of the time constants: *τ*_1_ was 13.8 s ± 10.1 for PKAR and 14.3 s ± 19.9 for vPKAR. The decay in the angular velocity had a mean *τ*_2_ of 153.1 s ± 112.1 for PKAR. Therefore, on the average, after 459.4 s ± 336.2 (3*∗τ*_2_) the posteffect of podokinetic stimulation vanished. The angular velocity of vPKAR decreased with a mean time constant (*τ*_2_) of 168.9 s ± 176.9. Therefore the posteffect of voluntary turning disappeared after 418.2 s ± 337.5. ANOVA showed no difference in the time course between the two conditions (*F*(1,15) = 0.2, *p* = 0.66). Both conditions collapsed, and there was a significant difference between the time constants of the increase in angular velocity (*τ*_1_) and of the vanishing of the posteffect (*τ*_2_) (*F*(1,15) = 36.6, *p* < 0.01). There was no interaction between time constants and conditions (*F*(1,15) = 0.16, *p* = 0.69). Further, for each subject, the time constants describing the time course of the vPKAR were plotted against the velocity during the corresponding conditioning procedure (Figure 2(c)). There was no relationship between *τ*_1_ and the angular velocity of voluntary turning (*R* ^2^ = 0.05, *p* = 0.4). However, the relationship between *τ*_2_ and the angular velocity of voluntary turning reached significance (*R* ^2^ = 0.25, *p* < 0.05), in spite of the large variability across subjects.

### 3.2. Time Lag between Head and Shoulder Movement

**Figure 3:**
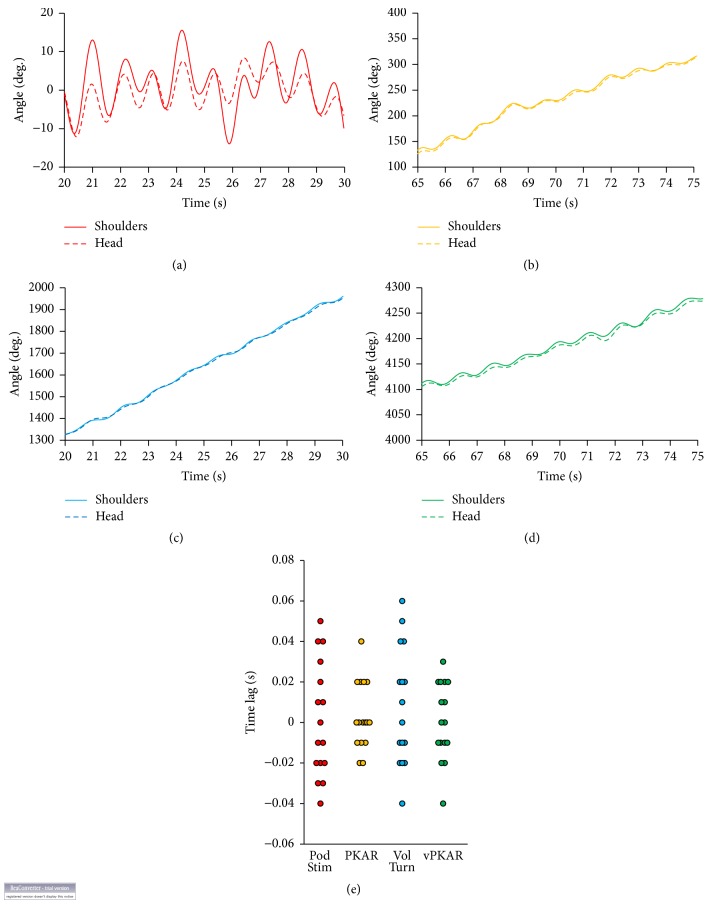
Coordinated head and shoulder movements. Angle described by the head (solid lines) and shoulders (dashed lines) mediolateral axis in a time period of 10 s during podokinetic stimulation (Pod Stim, (a)), PKAR (b), voluntary turning (Vol Turn, (c)), and its posteffect (vPKAR, (d)). During platform rotation, the body was kept almost fixed in space while head and shoulders showed minor left and right angular shifts. During voluntary turning (c) and during the two posteffects (b and d), head and shoulders continued to rotate in the horizontal plane, so that, in addition to their left and right yaw shift (a), the angle described by these segments continued to increase over time. The large differences in the* y*-scale amplitude between panel (a) and panels (b), (c), and (d) accommodate for the differences in the cumulative angle. The time lags between head and shoulders traces are reported in panel (e) for each subject. The 10 ms interval between the data points depends on the acquisition frequency; in many cases, several points coincide. There were no obvious differences across conditions.

The yaw angles described by head and shoulder axes of one subject during 10 s of podokinetic stimulation (a) and voluntary turning (c) and during the posteffects (PKAR (b) and vPKAR (d)) are reported in Figure 3. In (a), the traces are almost superimposable, indicating that head and shoulder girdle moved almost simultaneously. In order to estimate the time lag between head and shoulder angular rotation, cross-correlation analysis was performed on the traces of the yaw angle described by head and shoulder axes. Head and shoulder moved in phase under all conditions (*R* = 0.69 ± 0.16 for podokinetic stimulation and *R* = 0.90 ± 0.05 for PKAR; *R* = 0.92 ± 0.13 for voluntary turning and *R* = 0.94 ± 0.04 for vPKAR). Under all pre- and postconditions, shoulders and head moved almost simultaneously (Figure 3(e)) with time lags ranging across subjects and conditions from −40 ms to 60 ms. ANOVA showed no difference in time lag between voluntary turning and podokinetic stimulation (*F*(1,15) = 0.10, *p* = 0.75), no difference between pre- and posteffects (*F*(1,15) = 0.69, *p* = 0.42), and no interaction between conditions and pre- and posteffects (*F*(1,15) = 1.77, *p* = 0.2).

### 3.3. Head Acceleration

**Figure 4:**
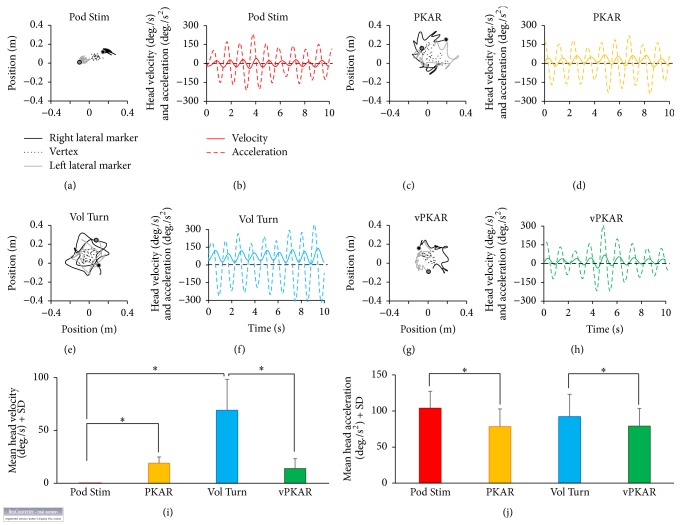
Head angular acceleration. (a) to (h) show the head movements in the horizontal plane during the podokinetic stimulation (Pod Stim, (a)), PKAR (c), voluntary turning (Vol Turn, (e)), and vPKAR (g). The two dots in (a), (c), (e), and (g) indicate the initial positions of the markers placed on the right (black dot) and left (grey dot) side of the head. Head velocity (solid line) and acceleration (dashed line) are reported in (b), (d), (f), and (h). The rotational effect of voluntary turning can be seen by the bias in the angular velocity profile (f). (i) and (j) show the mean values of head velocity (i) and acceleration (j) across subjects. Head showed angular acceleration (j) under both voluntary turning and podokinetic stimulation. There were no differences between the two posteffects for either head velocity or acceleration. *∗* indicates significant difference (*p* < 0.05) between mean head velocities (i) and accelerations (j).

The movement in the horizontal plane of the markers placed on right and left head side and on vertex and the head angular velocity and acceleration are reported in Figures 4(a)–4(h) for one subject. Data are reported for podokinetic stimulation (a and b), PKAR (c and d), voluntary turning (e and f), and vPKAR (g and h).

Across subjects, the mean head angular velocity (Figure 4(i)) was 69.1°/s ± 29.2 during voluntary turning and 0.01°/s ± 0.1 during podokinetic stimulation, while subjects tried to keep the head and trunk fixed in space. In the posteffects, head velocity was 18.9°/s ± 5.9 during PKAR and 13.9°/s ± 9.3 during vPKAR. ANOVA showed a significant difference in mean head angular velocity between conditions (*F*(1,15) = 40.64, *p* < 0.001) and a significant difference between pre- and posteffects (*F*(1,15) = 31.35, *p* < 0.001). There was a significant interaction between conditions (voluntary turning or podokinetic stimulation) and pre- and posteffects (*F*(1,15) = 227.38, *p* < 0.001). The head angular velocity was different between voluntary turning and podokinetic stimulation (post-hoc test, *p* < 0.001) but not between the two posteffects (post-hoc test, *p* = 0.17).

The mean angular acceleration of the head (computed on the rectified acceleration trace; see Section 2) was 103.8°/s^2^ ± 23.4 during podokinetic stimulation and 92.2°/s^2^ ± 30.8 during voluntary turning. In the posteffects, head acceleration decreased to 78.3°/s^2^ ± 24.5 for PKAR and to 79.1°/s^2^ ± 24.3 for vPKAR. ANOVA showed no difference in head acceleration between podokinetic stimulation and voluntary turning (*F*(1,15) = 1.45, *p* = 0.25). There was a difference between pre- and posteffects for the two conditions (*F*(1,15) = 24.8, *p* < 0.001), since head acceleration was smaller in the posteffect than during the conditioning period (post-hoc test, *p* < 0.05, for the two comparisons). There was no difference in head acceleration between the two posteffects (PKAR versus vPKAR: post-hoc test, *p* = 0.9). A comparison was also done between mean peaks of head acceleration values. The mean amplitude of the peaks was 158.8 ± 39.4°/s^2^ for podokinetic stimulation and 134.7 ± 44.3°/s^2^ for voluntary turning. During the two posteffects, the mean amplitude of the peaks decreased to 121.4 ± 38.6°/s^2^ for PKAR and to 118.8 ± 41.1°/s^2^ for the vPKAR. ANOVA showed a minor difference between conditions (*F*(1,15) = 4.01, *p* = 0.06) and a difference between pre- and posteffect (*F*(1,15) = 20.88, *p* < 0.001). There was no difference between the posteffects (post-hoc test, *p* = 0.8).

### 3.4. Cadence, Stance Period, and Height of Feet Lifting during Voluntary Turning and Podokinetic Stimulation and during Their Posteffects

**Figure 5:**
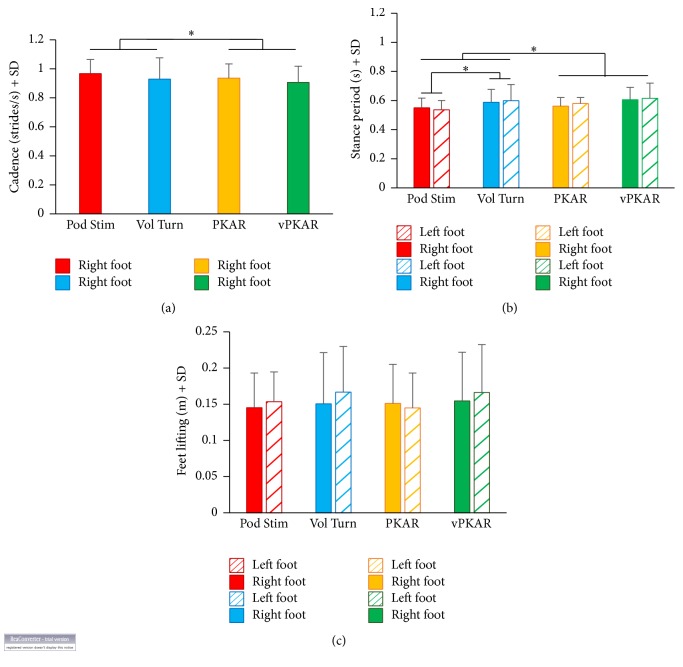
Cadence (a), duration of stance period (b), and height of feet lifting (c) during Pod Stim, Vol Turn, and the two posteffects. Cadence was not different between Vol Turn and Pod Stim but different between pre- and posteffects. The stance period (b) was somewhat longer during voluntary turning than during Pod Stim. Filled bars refer to the foot corresponding to the direction of rotation (right foot); striped bars refer to the foot opposite to the direction of rotation (left foot). There were no differences in height of feet lifting (c) between Vol Turn and Pod Stim or between the two posteffects. *∗* indicates significant difference (*p* < 0.05) between mean cadences (a) and mean stance period durations (b).

Figure 5 shows the mean cadence and mean duration of the stance period of stepping in place calculated in a time interval of 60 s during podokinetic stimulation and voluntary turning and around the time of the maximum velocity during the two posteffects. Mean cadence was 0.92 strides/s ± 0.14 during voluntary turning and 0.91 strides/s ± 0.11 in the posteffect. During the podokinetic stimulation, mean cadence was 0.97 strides/s ± 0.09 and 0.94 strides/s ± 0.1 during PKAR. There was no significant difference in cadence between voluntary turning and podokinetic stimulation (*F*(1,15) = 1.82, *p* = 0.19). The small difference in cadence between pre- and posteffect was significant (*F*(1,15) = 5.9, *p* < 0.05). However, the cadence during the posteffects in the 6 subjects turning at about the same velocities (mean velocity 65.26°/s ± 5.6) of the platform showed no significant difference between stimulation conditions (*F*(1,5) = 0.008, *p* = 0.93) and no difference between vPKAR and PKAR (post-hoc test, *p* = 0.2).

The stance periods were calculated for each foot during the podokinetic stimulation, the voluntary turning, and their posteffects. The mean stance period was slightly longer during voluntary turning than during podokinetic stimulation (*F*(1,15) = 5.33, *p* < 0.05). There was also a significant difference between voluntary turning or podokinetic stimulation and the two posteffects (*F*(1,15) = 16.52, *p* < 0.01), since the stance period was just longer during the posteffects, consistently with the slightly lower cadence.

The mean height of foot lifting during voluntary turning and podokinetic stimulation and during the two posteffects is reported in Figure 5(c). There were no significant difference between voluntary and podokinetic condition (*F*(1,15) = 0.9, *p* = 0.3) and no difference between pre- and posteffects (*F*(1,15) = 0.005, *p* = 0.95).

### 3.5. Step Angle and Rotation Velocity

**Figure 6:**
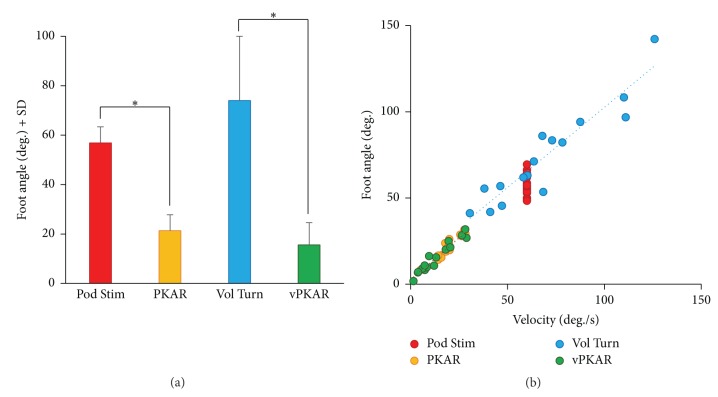
Foot angle (a) and its correlation with body rotation velocity (b). The mean foot yaw angle (a) between two consecutive stance phases was similar for podokinetic stimulation and voluntary turning. During the two posteffects, the mean angle diminished. In panel (b), the mean foot angle of each subject was plotted against the mean rotation velocity while stepping, for all conditions. There was a good relationship between foot angle and subject's angular velocity, both during voluntary turning and during the two posteffects. *∗* indicates significant difference (*p* < 0.05) between mean foot angles (a).

Figure 6(a) shows the mean foot angles calculated across subjects for each condition. The mean angle described by the foot on the horizontal plane during each step was 56.9° ± 6.5 during podokinetic stimulation and 21.4° ± 6.4 during the PKAR. The mean angle described by the foot during voluntary turning was 74.0° ± 27.5 and decreased to 15.6° ± 9.0 during the posteffect. ANOVA showed no significant difference between voluntary turning and podokinetic stimulation (*F*(1,15) = 1.35, *p* = 0.26). There were a significant difference between pre- and posteffects (*F*(1,15) = 226.87, *p* < 0.01) and an interaction between conditions and pre- and posteffect (*F*(1,15) = 29.14, *p* < 0.01), since foot angle diminished slightly more during the posteffect of the voluntary turning than during the PKAR, in compliance with the overall slower turning velocity.  Figure 6(b) shows the mean foot angle for each subject in each condition plotted against the mean angular velocity of the shoulders. There was a good relationship between the angle described by the foot and the subject velocities during both voluntary turning (*R* ^2^ = 0.87, *p* < 0.01) and the posteffects (vPKAR: *R* ^2^ = 0.94, *p* < 0.01, PKAR:*R* ^2^ = 0.9, *p* < 0.01).

## 4. Discussion

Turning around while stepping in place can be produced voluntarily or be the consequence of a stimulation applied during the stepping task, such as axial muscle unilateral vibration [22, 23, 30] or vestibular [31, 32] or optokinetic stimulation [25, 33]. One elegant way of producing inadvertent turning around while stepping in place eyes closed is to “induce” this behavior by having subjects stepping on a rotating platform while maintaining fixed heading by referencing body orientation to the seen environment [14, 20, 34]. In the literature, the platform training produces the podokinetic stimulation while the posteffect is called the podokinetic adaptation (podokinetic after-rotation, PKAR) [14, 18–20].

We tested the hypothesis that prolonged voluntary stepping in place while deliberately turning around on a motionless floor also produces a posteffect similar to PKAR. Hence, we sought an answer to the following questions: can a rotatory posteffect be produced by continuous, deliberate whole-body turning around while stepping (henceforth voluntary turning), as well as by the podokinetic stimulation consisting in a repeated displacement of feet orientation by a rotating platform (in turn counteracted by replacing the foot in a position compatible with heading maintenance)? If so, are there differences between the “true” PKAR and the posteffect of voluntary turning (vPKAR) in spite of the differences between the tasks? Can this comparison tell us something about the mechanisms underpinning PKAR?

At first sight, it would seem odd enough that a deliberate motor behavior contains in itself the potential for its inadvertent persistence after the termination of the specific voluntary command, not least, because of the presence of the efference copy during voluntary movement, which tends to cancel the feedback sensory information from the moved segments [35, 36]. However, examples are available for after-effects of deliberate motor actions, which normally tend to show features germane to the pristine task. For instance, a strong isometric effort of shoulder muscles for upper limb abduction, as by counteracting someone else's push against the hand for half a minute or so, produces an involuntary arm elevation (the Kohnstamm phenomenon) ([37] and see [38]) that inadvertently ensues at the end of the voluntary contraction. Further, walking on a linear treadmill, at variance with overground walking, produces an after-effect on the orientation of the standing body in the form of a forward body inclination lasting for a few minutes [39]. In this case, body inclination would be the consequence of a change in the postural reference arising from treadmill locomotion itself, possibly connected to the peculiarities of this type of walking [40]. Moreover and more interestingly for the present account, when standing subjects oppose for a while a rotational torque applied to the pelvis, an involuntary postcontraction of the trunk muscles is observed. If these subjects start walking, they walk along a curved trajectory in the direction of the preceding torsion [41].

Hence, a posteffect of prolonged stepping in place while deliberately turning around may not be beyond belief. We show here that the posteffect of voluntary rotation (vPKAR) shares all the features of the PKAR induced by the podokinetic stimulation while stepping on the rotating platform. This applies to the evolution of the turning velocity over time, including rising time, peak velocity, and the decay after that peak. Indeed, no significant difference exists in the rise and decay time constants of the turning velocity profile between PKAR and vPKAR posteffects. Of course, larger interindividual differences were observed under the voluntary turning condition than under the podokinetic stimulation, since in the latter case the turning velocity was fixed, while in the former it was self-paced. Interindividual differences could also be observed during the posteffects. However, interestingly, the almost fixed ratio of the turning velocity post- (PKAR or vPKAR) versus prerotation (podokinetic stimulation or voluntary turning) was analogous, both within and across subjects, even if it is somewhat smaller for the vPKAR (about 20%) compared to PKAR (about 30%). This small difference observed in the grand means was reproduced when the posteffects were compared within a smaller subject subgroup for which the velocities were nondifferent during podokinetic stimulation and voluntary turning. Therefore, differences between voluntary turning (eyes closed) and platform stimulation protocols (eyes open) can affect, albeit to a limited extent, the process whereby the central nervous system integrates the podokinetic stimulation and sets the rotation velocity in the postperiod.

An apparently important difference between the two conditioning procedures is that vision was banned under the voluntary turning condition, which was performed being blindfolded. However, based on the similar quality of the posteffects, one would deduce that vision (a quasi-stable visual field is available* during* the podokinetic stimulation on the rotating platform) may not intrude at all in the acquisition of the PKAR features. As a limitation, we would note that our procedure did not allow assessing any effect of vision on the PKAR itself, since the podokinetic stimulation was always administered eyes open. This was necessary in order to have a constant orientation in space of the subjects, so that their feet and leg rotations counteracted closely the platform rotation. In other cases, the constant orientation in space was obtained by having subjects holding onto a hearth-fixed rail [17, 20, 26], but in no case were PKARs compared between podokinetic stimulations with and without vision. The role of vision may not be dissimilar to what happens while walking on the linear treadmill, where a mismatch also exists between the quasi-stable visual field and the “expected” visual flow connected to the virtual body progression. Yet, availability or suppression of vision has no major effects on dynamic stability in treadmill walking [42]. In passing and by necessity, vision has no effect on the PKAR itself or on the vPKAR, since eyes were closed in both tasks.

A vestibular input is certainly elicited by turning around the vertical axis [43] and it interacts with the generation of the PKAR [44]. The vestibular input could differ between voluntary turning and podokinetic stimulation and contribute to the differences observed in the peak velocity of vPKAR and PKAR. In the former case, during conditioning, the head undergoes a yaw rotation at about the same velocity and direction as the rotating body, while in the latter head and body do not quite rotate, unless for the minor yaw movements associated with the side-to-side displacement of head and trunk accompanying the stepping task. However, the mean angular head* acceleration* in the yaw plane proved to be not different during the voluntary rotation and the stepping on the rotating platform, since the value of the angular acceleration is mainly dependent on the small but fast horizontal to-and-fro yaw rotations of the head mentioned above. The ample but slow head rotations accompanying whole-body rotation while voluntarily stepping in place and turning around are relatively smooth and add little to the peak values of acceleration. Thus, the vestibular inflow may be comparable, under steady state, between the two conditioning procedures. However, Earhart et al. [27] have shown that the PKAR is somewhat enhanced in bilateral vestibular loss patients, suggesting an inhibitory action of the normal vestibular input on PKAR velocity. In this light, one might suppose a stronger vestibular input for voluntary turning compared to podokinetic stimulation, to account for the differences mentioned above. Indeed, the vestibular input must be stronger* just before* vPKAR compared to PKAR, since head velocity undergoes large changes at the end of the voluntary rotation (see Figure 1(d)). Instead, almost no changes are observed at the end of the podokinetic stimulation (see Figure 1(c)), because the head angular rotation was almost nil during the platform rotation (compare the angular velocity profiles in Figures 4(b) and 4(f)). Hence, the phasic vestibular stimulation occurring at the end of voluntary rotation could have prevented the peak rotation velocity of vPKAR to reach that of PKAR.

The global kinematic organization of the turning behavior in the posteffects was superimposable. The cadence of the stepping task, the duration of the stance period, and the height of feet lifting were not significantly different between the posteffect of podokinetic stimulation and that of voluntary turning. A strong relationship also appeared between amplitude of foot angular rotation and velocity of whole-body rotation, which somehow trivially shows that body rotation is dictated by the value of foot yaw rotation in the successive stance phases. This relationship held under both conditions, and its value was superimposable between the two posteffects. Also, the time lags between the periodic yaw rotations of shoulder and head were fully superimposed. Hence, both PKAR and vPKAR are characterized by* en *blochead rotation and trunk rotation, in turn not different from that occurring during voluntary turning or stepping on the rotating platform. This conforms to the suggestion by Earhart and Hong [26], which advocated that PKAR is relayed through the same locomotor circuits active at the beginning of voluntary turning. Among the similarities of PKAR and vPKAR, it is important to mention that, under both voluntary turning and podokinetic stimulation conditions, the postrotation took place without the least perception of it by any subject, in spite of the yaw rotation velocity being certainly above the vestibular perception threshold at the yaw periodic rotations of about 1 Hz (or the stepping cadence) [45, 46]. Subjects themselves were startled when, well after their performance, they observed their final position and were told that that position was the final outcome of several spin movements.

During the podokinetic stimulation, feet (*and* legs) are passively rotated when they are placed on the platform during the stance phase, and subjects look at the surrounding environment that is their reference for keeping head and trunk almost fixed in space. Instead, during voluntary stepping and turning around, the supporting floor is stationary and the stance foot and leg are stable (they rotate during the swing phase), and trunk and head do rotate in space. A key event common to the two conditions is the rotation of the pelvis on the stance foot. During the voluntary rotation, one rotates the leg with respect to the pelvis (i.e., an external rotation of the leg, in the direction of the intended turning around) in the swing phase of stepping and rotates the pelvis pivoting on the stance foot in the same direction relative to space (i.e., an “active” internal rotation of the leg) during the stance phase. Conversely, during the platform rotation, the foot is being “passively” extrarotated in the direction of the platform rotation for the stance period, while the trunk rotates onto it for holding its orientation in space.

Hence, the truly common event to podokinetic stimulation and voluntary rotation is the active rotation of the trunk on the stance foot. This certainly requires the production of a nonnegligible force in the pelvic muscles that rotate internally the thigh, in order to have the heavy trunk keeping the pace with the foot extrarotation that has occurred during the immediately earlier swing phase (a task requiring minimal force). In this light, both PKARs would be produced by the voluntary effort of rotating the trunk on the feet, much as a voluntary deltoid effort produces the Kohnstamm arm-raising phenomenon or a voluntary trunk rotation effort produces a curved trajectory during a subsequent locomotion task [41]. Of note, a prolonged static twist of the trunk on the feet of 30° around the vertical axis during stance, maintained for 10 minutes, induced a subsequent unperceived postural reorientation but induced no PKAR when subjects were asked to step in place [34]. This indicates that PKAR is not an automatic consequence of postural reorganization but likely depends on the presence of a continuous motor output during the stepping condition [34]. The passive external rotation produced by the rotating platform would be the counterpart of the active (minimal-effort) external rotation during the swing phase in the voluntary rotation. In both cases, this extrarotation produces another event, that is, the passive stretch of the leg-intrarotating muscles. All in all, one might argue that the PKAR and the vPKAR rest both on the periodic active contraction of the lower limb intrarotating muscles, followed by their periodic passive stretch. The shortening-stretch cycle would appear the appropriate event, necessary for triggering an effective discharge in the muscle spindles, able to enhance the excitability of the locomotor circuits normally subserving turning (see [38], for a discussion of peripheral models of the Kohnstamm phenomenon generation).

We would only briefly speculate about the brain circuits that are potentially involved in the generation of PKAR and vPKAR in response to the periodic proprioceptive afferent volley from the muscles rotating the lower limb. Of note, during the Kohnstamm movement there is widespread activation of the cerebral cortex [47], and during the PKAR subjects show a direction-specific deviation of the subjective straight-ahead [48]. Therefore, the cerebral cortex, most likely the posterior parietal cortex, must be involved in the body orientation occurring during PKAR and vPKAR. In this light, subjects would trail their modified subjective straight-ahead while stepping in place, much as what occurs during neck muscle vibration [30], which is also known to affect the straight-ahead [49]. Courtine et al. [23] showed that axial but not appendicular muscle vibration produces a clear-cut deviation of the locomotor trajectory. In this vein, we would consider the (axial) pelvic muscles an important source of the proprioceptive input playing a role in the PKARs.

We would also note that the sum of the vestibular input and the proprioceptive input from the pelvis muscles might have affected the PKARs [50], much as what occurs for the sum of vestibular and neck input in the definition of the yaw motion perception [24]. In the cases mentioned in Pettorossi et al. [51, 52], the changes in the abnormal perception after a prolonged rotational vestibular stimulation and neck vibration are long-lasting. That duration is of the same order of magnitude as the duration of the PKARs, pointing to a central modulation of the deviated perception of the straight-ahead in both cases (see [24] for a review). We would also point out that while the repeated muscle contraction would create the background for a posteffect, the particular sequence of the active stepping movements would confer the peculiar rhythmic features to the posteffects. It is as if the build-up of the charge of the “integrator battery,” wherever and whatever it is, contains in itself the memory of the pattern responsible for the process.

On the translation to the clinical side, this line of research goes in the direction of recent findings showing the relevance of practicing a specific task with added challenges to a training regimen ([53], in rats). Stepping in place while voluntarily turning can substitute and be effective almost as much as the rotating platform for training of the turning coordination [29, 54] in patients with impairment of turning synergies of various origin [9–11, 16, 55, 56].
